# Occult cirrhosis presenting as progressive tremor: acquired hepatocerebral degeneration induced by traditional Chinese medicine and natural health products, aggravated by manganese supplements: A case report

**DOI:** 10.1097/MD.0000000000048686

**Published:** 2026-05-08

**Authors:** Chuan Liu, Roumin Wang, Xiaoyan Li, Wang Ni, Gonglu Liu, Yi Dong

**Affiliations:** a Department of Medical Genetics and Centre for Rare Diseases, Second Affiliated Hospital, Zhejiang University School of Medicine and Zhejiang Key Laboratory of Rare Diseases for Precision Medicine and Clinical Translation, Hangzhou, Zhejiang, China.

**Keywords:** acquired hepatocerebral degeneration, herb-drug interactions, hypermanganesemia, natural health products, occult cirrhosis, psoralea corylifolia, traditional Chinese medicines

## Abstract

**Rationale:**

Acquired hepatocerebral degeneration (AHD) is a rare neurological complication of chronic liver disease or portosystemic shunting, typically characterized by extrapyramidal symptoms and manganese accumulation in the basal ganglia. Its diagnosis is particularly challenging in patients with occult cirrhosis and complex exposure histories, especially when traditional Chinese medicines (TCM) and natural health products (NHPs) contribute to hepatotoxicity.

**Patient concerns:**

A 66-year-old woman presented with a 2-year history of progressive postural and intention tremor, with marked worsening over the preceding 2 months, interfering with daily activities.

**Diagnoses:**

Laboratory tests indicated hepatic dysfunction and cirrhosis, while neuroimaging revealed bilateral T1 hyperintensity in the basal ganglia. Extensive evaluation excluded viral hepatitis, alcohol-related liver disease, autoimmune liver disease, Wilson disease, and occupational toxic exposure. Whole-blood manganese was markedly elevated. Detailed history-taking revealed long-term use of hepatotoxic TCM (notably *Psoralea corylifolia*) and manganese-containing supplements. A diagnosis of manganese-related AHD secondary to cirrhosis, likely induced by chronic TCM/NHPs exposure, was established.

**Interventions:**

Management included discontinuation of hepatotoxic agents and manganese-rich supplements, implementation of a low-manganese diet, hepatoprotective therapy, ammonia-lowering treatment, symptomatic control of tremor (clonazepam and trihexyphenidyl), and chelation therapy with dimercaptosuccinic acid.

**Outcomes:**

At follow-up, liver function and ammonia levels improved, whole-blood manganese levels decreased but remained elevated, and tremor severity was significantly reduced with corresponding functional improvement.

**Lessons:**

This case highlights that AHD may arise in the setting of occult cirrhosis related to long-term TCM/NHPs use, with additional manganese exposure acting as a precipitating factor. Clinicians should routinely screen for liver dysfunction in patients with unexplained extrapyramidal symptoms, incorporate manganese testing and basal ganglia magnetic resonance imaging into diagnostic evaluation, and systematically inquire about TCM/NHPs use. Multidisciplinary management is crucial, particularly in regions with widespread use of TCM and NHPs.

## 1. Introduction

Acquired hepatocerebral degeneration (AHD), also known as non-Wilsonian hepatocerebral degeneration, is a chronic neurological syndrome observed in advanced hepatobiliary disease or portosystemic shunting. Its prevalence is estimated to be 1 to 2% among patients with cirrhosis. Currently, the most common causes are chronic viral hepatitis, alcohol-related liver disease, metabolic dysfunction-associated steatotic liver disease, and autoimmune hepatitis.^[1,2]^ As long as there is long-term liver dysfunction or collateral shunting between the portal and the systemic system, toxic substances can bypass the liver and deposit in the brain and trigger oxidative stress and inflammation.^[1,3]^ Patients with AHD often present with insidious and progressive extrapyramidal symptoms. Manganese plays a very important role in neurotoxicity. Typical magnetic resonance imaging (MRI) shows bilateral T1 hyperintensity of the globus pallidus and adjacent basal ganglia.^[4,5]^

Traditional Chinese medicines (TCM) and natural health products (NHPs) regimens are well-documented causes of hepatotoxicity and drug-induced liver injury.^[6]^ Common phenomena such as unregulated composition, adulteration and herb-drug interactions increase the risk of hepatotoxicity.^[7]^ In patients with DILI progressing to cirrhosis, impaired biliary manganese excretion permits systemic accumulation and cerebral deposition.^[1]^

We report a case of AHD in an elderly woman whose clinical, biochemical and imaging profiles supported manganese-related AHD in the setting of occult cirrhosis likely attributable to chronic TCM and NHPs exposure. Through this case, we emphasize that clinicians encountering extrapyramidal symptoms must proactively screen for occult liver disease and blood manganese levels, and routinely inquire about the use of TCM and NHPs. In addition, clinicians should strengthen their recognition of the imaging features characteristic of AHD, such as bilateral globus pallidus T1 hyperintensity.

## 2. Case presentation

A 66-year-old female farmer who had spent her entire life in rural Zhejiang province presented to our clinic with a complaint of tremor persisting for more than 2 years, which had worsened during the past 2 months. The tremor had begun insidiously: 2 years earlier she noticed subtle shaking of her hands, particularly evident when reaching for objects or maintaining a posture. The tremor progressed so slowly that it was initially attributed to aging or fatigue. Several months later, the patient herself even felt that the symptoms had become so insignificant that they seemed to have disappeared. She was able to continue the farmwork and household. Over the last 2 months before admission, the tremor accelerated in amplitude and interfered with daily tasks (eating, getting dressed and carrying water) and prompted her to seek neurologic evaluation.

**Figure 1.**
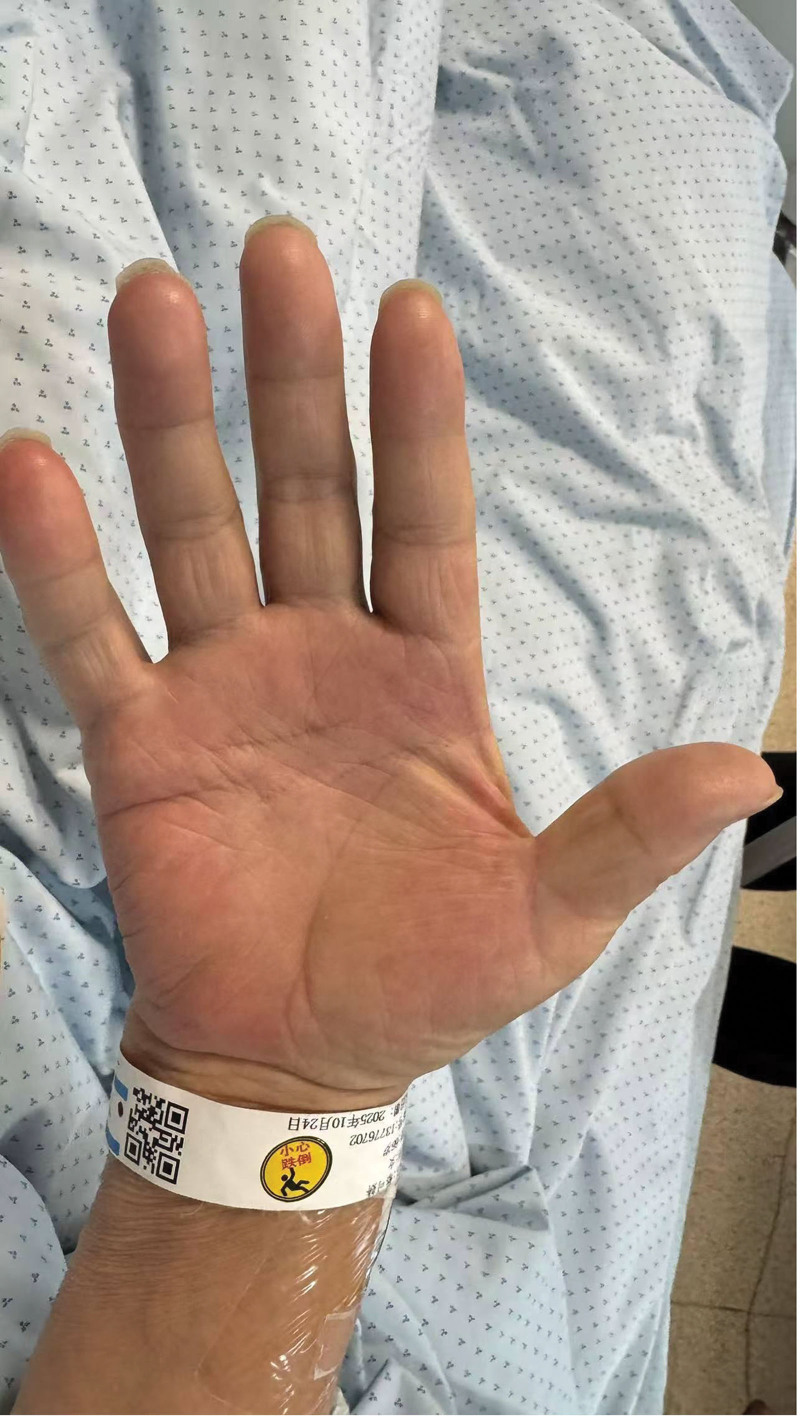
The palmar erythema of the patient.

On admission the neurological examination demonstrated increased muscle tone in all 4 limbs, bilateral postural and intention tremor more pronounced on the left side (Video 1), palmar erythema (Fig. 1), and a positive right Babinski sign. There were no resting tremors typical of Parkinson disease. Slit-lamp examination revealed no Kayser–Fleischer rings. No additional abnormalities were found on neurological examination.

**Video 1.** 

In the initial laboratory tests, signs of liver dysfunction and cirrhosis were already evident. Liver function tests: aspartate aminotransferase 54 U/L↑ (reference range 13–35 U/L), gamma-glutamyl transpeptidase 57 U/L↑ (reference range 7–45 U/L), alanine aminotransferase 29 U/L (reference range 7–40 U/L), total bilirubin 23.1 μmol/L↑ (reference range < 21 μmol/L), direct bilirubin: 5.9 μmol/L↑ (reference range < 4 μmol/L), indirect bilirubin: 17.2 μmol/L↑ (reference range 5–20 μmol/L) and ammonia 66.4 μmol/L↑ (reference range 9–33 μmol/L). Liver synthetic function revealed albumin: 35.1g/L (reference range 35–52 g/L), globulin: 30.8g/L↑ (reference range 15–30 g/L), albumin/globulin ratio: 1.14↓ (reference range 1.2–2.4) and a prolonged prothrombin time (14.4 seconds↑, reference range 11–14 seconds). Serum markers of liver fibrosis were elevated, including type III procollagen peptide (65.03 ng/mL, reference range < 30 ng/mL), type IV collagen (95.42 ng/mL, reference range < 30 ng/mL), laminin (62.78 ng/mL, reference range < 50 ng/mL), and hyaluronic acid (298.19 ng/mL, reference range< 100 ng/mL).

Serological markers for viral hepatitis were all negative, including hepatitis A virus IgM antibody, hepatitis B surface antigen, hepatitis B surface antibody, hepatitis B e antigen, hepatitis B e antibody, hepatitis B core antibody, hepatitis C virus antibody, and hepatitis E virus IgM antibody. Workup for Wilson disease was normal: ceruloplasmin 256 mg/L (reference range 200–600 mg/L) and 24-hour urine copper 12.6 μg/24 hours (reference range 15–60 μg/24 hours). Autoimmune liver disease serologic markers were all negative, including anti-mitochondrial antibody type 2, anti-liver kidney microsomal antibody, anti-liver cytosol antibody, anti-soluble liver antigen/liver-pancreas antibody, anti-gp210 antibody, and anti-sp100 antibody. Serum trace elements including calcium, iron, zinc, copper, and magnesium were within normal limits. Tumour markers revealed no abnormalities: carcinoembryonic antigen was 1.2 ng/mL (reference < 5 ng/mL), alpha‑fetoprotein was 2.0 ng/mL (reference < 20 ng/mL), and carbohydrate antigen 199 was 5.1 U/mL (reference < 37 U/mL). Serum M protein was undetectable by electrophoresis. Extended laboratory and ancillary investigations, including glycated hemoglobin, human cytomegalovirus and Epstein-Barr virus DNA testing, serum lactate, bone metabolism markers, anemia panel, urinalysis with urinary sediment quantification, stool examination, HIV and syphilis serology, thyroid function tests, revealed no abnormalities.

**Figure 2.**
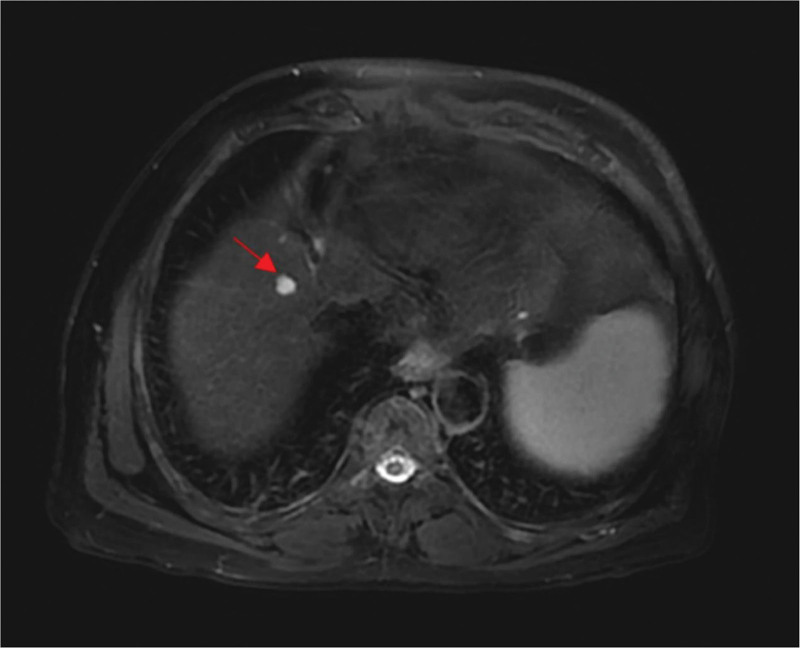
The contrast-enhanced liver MRI showing the patient’s HGDN. HGDN = high-grade dysplastic nodule, MRI = magnetic resonance imaging.

Hepatic imaging clearly and consistently revealed cirrhosis with portal hypertension. Contrast-enhanced computed tomography showed a cirrhotic liver, splenomegaly, gastroesophageal varices and portosystemic shunting. Next, we conducted liver magnetic resonance and stiffness measurement. The results showed liver stiffness in the F4 stage (indicating liver cirrhosis), an enlarged spleen, and small nodules in segment VIII of the liver, which suggested a possibility of small hepatocellular carcinoma. Therefore, we performed contrast-enhanced liver MRI scanning, the results of which indicated that the small nodule in segment VIII of the liver was considered as a high-grade dysplastic nodule (HGDN) (Fig. 2).

**Figure 3.**
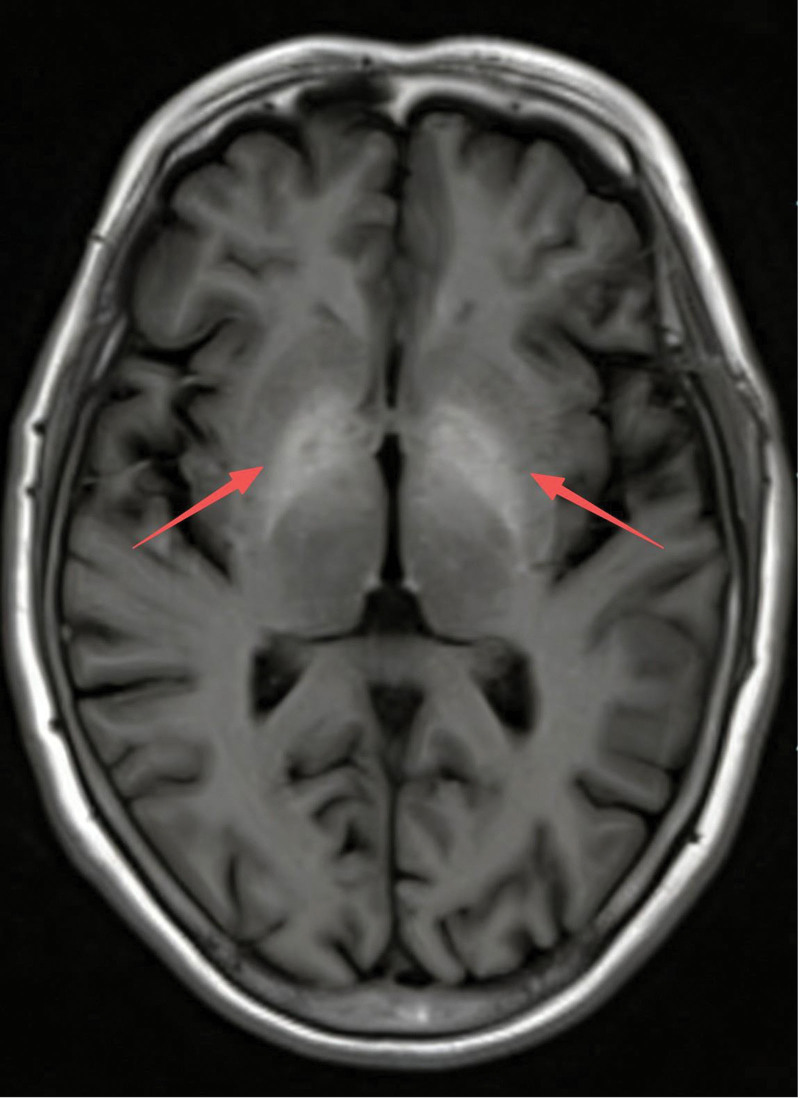
The brain MRI demonstrated bilateral T1 hyperintensity of the globus pallidus and posterior limb of the internal capsule. MRI = magnetic resonance imaging.

Neuroimaging and electrophysiology have also confirmed typical abnormalities. Brain MRI demonstrated bilateral T1 hyperintensity of the globus pallidus and posterior limb of the internal capsule (Fig. 3), with small hemorrhagic foci in the left frontal lobe and right cerebellum demonstrated by susceptibility-weighted imaging. Tremor was confirmed by electromyographic recording, which demonstrated pathological tremor activity in the left upper limb. During the examination, the overall tremor amplitude showed a progressive increasing trend. Under slight loading, the agonist and antagonist muscles exhibited synchronous activation with a monophasic burst pattern. During rest and postural conditions, tremor discharges remained monophasic, a pattern compatible with postural and intention tremor rather than the primary resting tremor of Parkinson disease.

Because the patient was confirmed to have the aforementioned liver cirrhosis, and the head MRI T1 hyperintensity in the basal ganglia also indicated the manifestations of liver-originated brain damage,^[8]^ we conducted a more comprehensive test for blood trace elements. After the results were returned, a significant anomaly emerged. The whole-blood manganese level was 40.9 μg/L (reference range 4.7–18.3 μg/L), more than twice the upper limit. The blood levels of lead, copper, iron and zinc were within reference ranges. Based on the markedly elevated blood manganese level, we reinterviewed the patient and her family regarding any occupational history as a welder, miner, steelworker, or employment in industries with heavy-metal exposure, as well as possible involvement in construction or renovation work. We also inquired about potential environmental toxic exposures related to her residence and surroundings. All were denied.

Her medical history was notable for a diagnosis of multiple myeloma (MM) made 5 years earlier after repeated episodes of epistaxis. She completed a full course of lenalidomide therapy that stopped in January 2025 in compliance with the Haematologist’s instructions. Suspected adverse hepatic reactions have been reported in < 1% of patients treated with lenalidomide, most commonly abnormal liver function tests or clinical signs of hepatic disorders, and rarely hepatic failure with an intermediate onset of 7–10 days after initiation, which indicates that even when lenalidomide causes hepatic injury, it is typically acute rather than chronic. In reported cases, discontinuation of therapy has typically resulted in normalization of liver function within 2–3 weeks (range: 8–30 days).^[9,10]^ She denied alcohol use or smoking. Her body mass index was 22.31, which was within the normal range. Among individuals in her surrounding environment with similar living conditions and close contact, no comparable symptoms were observed.

When the diagnosis became challenging and the etiology of cirrhosis remained unclear, we decided to conduct a more detailed history taking. Upon that, an essential medical history, specifically a long-term lifestyle, was elicited. This elderly woman had a long-standing adherence to TCM. For over a decade, she had repeatedly sought care at local TCM shops for chronic complaints she referred to as “internal heat.” She described her symptoms as nonspecific discomforts, including dry mouth, halitosis, constipation, and fatigue. She interpreted this condition as a state of hyperactivity of fire, or “shanghuo,” a TCM concept describing excessive “yang” activity (associated with heat, fire, and positivity). She routinely collected herbal formulas and wild plants by herself (often several times per week). However, the patient had no prior liver-related symptoms or abnormal findings. Her only previous health examination, conducted more than a decade ago, revealed normal liver enzyme levels and unremarkable hepatobiliary and splenic ultrasound results.

After MM diagnosis, she increased the use of TCM and NHPs recommended locally. She specifically prescribed a series of TCM aimed at strengthening bone function. Many of the TCM and NHPs previously used by the patient could not be recalled. However, by combining the patient’s and her family’s recollections, several potentially culpable ingredients were identified. *Psoralea Corylifolia* (Buguzhi, literally “bone-strengthening resin” in Chinese) is one of the most commonly used medicinal plants in TCM and has been reported to exert effects in MM, such as inducing apoptosis in MM cells.^[11]^ However, several studies have demonstrated that this component causes definite hepatotoxicity.^[7,12]^ Her TCM prescription also included Fuzi (*Aconitum carmichaeli* Debxi), which has been reported by some researchers to be toxic.^[13]^ Diester-diterpene alkaloids, which are toxic substances mainly owing to their effects on voltage-dependent sodium channels, are also included in her medication list.^[14]^ The patient reported that after taking that series of TCM aimed at strengthening bone function, sleep difficulties began, which may be related to diester-diterpene alkaloids. She also habitually consumed dried black fungus (*Auricularia auricula*) and various seafood products, including daily consuming of dried sea cucumber (*Stichopus* spp.). She believed that these foods could strengthen her body following the diagnosis of MM. These items are known to be rich sources of manganese.^[15,16]^

It is noteworthy that the patient’s chief complaint included worsening tremor over the past 2 months. Two months earlier, she experienced knee discomfort and was diagnosed at another hospital with degenerative changes in the knee joint and left knee effusion, for which joint aspiration was performed. Subsequently, she self-purchased a multicomponent joint/cartilage supplement (the exact composition cannot be specified by the patient), but commercially available joint health formulations are typically multicomponent products that include glucosamine, chondroitin, calcium, and trace minerals such as manganese. For example, Glucosamine MAX (Herbs of Gold, Australia) and Glucosamine Chondroitin (Nutra-Life, New Zealand) both list manganese among their ingredients. Following supplementation, she noted her hand tremor had worsened compared with 2 months earlier, prior to starting the joint product.

The patient’s history of chronic TCM and NHPs exposure, objective evidence of cirrhosis with portosystemic shunting, MRI pallidal T1 hyperintensity, and markedly elevated whole-blood manganese level were taken into account. Differential diagnoses such as viral hepatitis, alcoholic cirrhosis, autoimmune liver disease, and iron overload, were excluded. Wilson disease was carefully considered as a differential diagnosis, given the cirrhosis and overlapping extrapyramidal manifestations. However, ophthalmological examination did not reveal Kayser–Fleischer rings, and brain MRI lacked the classical symmetric T2 hyperintensities described in Wilson disease.^[8]^ These results, combined with normal ceruloplasmin and urinary copper excretion, allowed us to confidently exclude Wilson disease. As genetic testing did not confirm biallelic ATP7B mutations (which was subsequently verified by later genetic testing results), the diagnosis of Wilson disease requires either decreased serum ceruloplasmin and/or elevated 24‑hour urinary copper excretion, or the presence of Kayser–Fleischer rings; none of these criteria were met in our patient. Regarding the tremor mentioned above, our patient exhibited postural and intention tremor rather than classic parkinsonian features. As reviewed by Shin and Park,^[1]^ rest tremor is rarely observed in parkinsonism related to AHD and is usually accompanied by postural or action tremor in 1 or both hands.

Based on the diagnosis, management was organized into several components: advising the patient to discontinue potential hepatotoxic exposures and a low-manganese diet, improving hepatic function, managing neurological symptoms, and initiating chelation therapy to reduce manganese overload. Specifically, management included the use of bicyclol tablets and polyene phosphatidylcholine capsules to reduce hepatic enzyme levels, and L-ornithine L-aspartate injection to lower blood ammonia level. Clonazepam (0.5 mg orally every night) and trihexyphenidyl hydrochloride (1 mg orally twice daily) were administered to alleviate hand tremors. Dimercaptosuccinic acid (DMSA) capsules (in the first month, the patient took 0.25 g orally twice daily. In the second month, the dosage was increased to 0.5 g orally twice daily) were employed for manganese chelation therapy; this agent is routinely used by our team as an oral heavy-metal chelator in Wilson disease patients, with extensive experience in such applications. Supportive care focused on maintaining the electrolyte balance, managing osteoporosis, and improving sleep quality.

No family history of similar disorders was reported, and no related symptoms were observed among her family members. Whole-exome sequencing was performed; however, no pathogenic or likely pathogenic variants consistent with the patient’s clinical phenotype were identified. For the patient’s HGDN, we consulted both the gastroenterologist and the hepatobiliary surgeon regarding potential interventions such as liver biopsy, surgical resection, and thermal ablation. After discussion with the patient and family, the final conclusion was to defer these interventions because the patient had normal tumor markers, including carbohydrate antigen 199 and carcinoembryonic antigen. Both specialists considered that the risk of malignant transformation of the HGDN at this stage was not particularly high and recommended conservative management with regular follow‑up instead. In addition, there was concern about potential procedure‑related complications. Accordingly, the patient was advised to return for follow-up within 1 month, with close surveillance at shorter intervals during the subsequent period. If the condition remained stable, the follow-up interval was gradually extended. At the 2-week follow-up after initiation of treatment, laboratory parameters demonstrated partial biochemical improvement: aspartate aminotransferase decreased from 54 U/L to 43 U/L (reference range 13–35 U/L), alanine aminotransferase from 29 U/L to 27 U/L (reference range 7–40 U/L), total bilirubin from 23.1 μmol/L to 16.5 μmol/L (reference range < 21 μmol/L), direct bilirubin from 5.9 μmol/L to 0.1 μmol/L (reference range < 4 μmol/L), and indirect bilirubin from 17.2 μmol/L to 14.2 μmol/L (reference range 5–20 μmol/L). Serum ammonia levels also declined from 66.4 μmol/L to 51.5 μmol/L (reference range 9–33 μmol/L). At the second-month follow-up, the whole-blood manganese level decreased from 40.9 μg/L to 22.8 μg/L (reference range 4.7–18.3 μg/L), but remained elevated. Tremor severity showed marked clinical improvement at follow-up, with reduced amplitude on neurological examination. The patient also reported subjective improvement in daily activities.

## 3. Discussion

This case represents a challenging and instructive example of AHD because the patient did not present with manifestations of liver disease, such as jaundice or ascites, her liver disease remained “hidden,” as occult cirrhosis rather than a long-standing cirrhotic history typically observed in most cases of AHD. Furthermore, the absence of alcohol use, viral hepatitis, autoimmune liver disease, and occupational heavy-metal exposure further exacerbated this occult presentation. The presentation can be regarded as an acute-on-chronic event, in which the patient’s self-purchased manganese-containing joint supplement 2 months earlier pushed the patient beyond the threshold, precipitating the current admission, although such intake is typically tolerated in healthy individuals. A general neurologist without specifically ordering the hepatic examination might reasonably misdiagnose the condition as essential tremor or Parkinson disease. As a tertiary center specializing in rare neurological disorders, our diagnostic protocol involves careful differentiation of basal ganglia imaging abnormalities. When a hepatic etiology is suspected, we have sufficient investigative modalities and clinical expertise to evaluate liver imaging, enabling us to establish the correct diagnosis.^[17,18]^

Long-term exposure to TCM and NHPs is considered a principal factor in the etiology of cirrhosis in this patient. Several key findings supported this conclusion. First, the patient had normal liver enzymes and normal hepatobiliary ultrasonography findings more than a decade earlier, making congenital or early-onset metabolic liver diseases unlikely. Second, she had no history of alcohol consumption, viral hepatitis, autoimmune disease, obesity, or metabolic syndrome, and no family history suggestive of inherited disorders. In contrast, she reported frequent and sustained use of TCM and self-collected herbal products for more than 10 years, with a marked increase after the diagnosis of MM. Among the identified components, *P. corylifolia* has been repeatedly reported to cause hepatotoxicity. Gao et al demonstrated dose-related hepatotoxicity of *P. corylifolia* extracts in a zebrafish model, mediated by inhibition of farnesoid X receptor and peroxisome proliferator-activated receptor alpha expression, leading to bile acid and lipid metabolism disorders.^[19]^ In addition, there was temporal consistency between her sleep disturbance and the use of Fuzi (*A. carmichaeli* Debx). Moreover, the cumulative burden of multi-ingredient, nonstandardizedstandardized herbal formulations and wild plants further increased the risk of drug-induced liver injury.^[11,20,21]^ The temporal relationship between intensified herbal/NHPs use and the emergence of liver dysfunction strongly favors an acquired toxic etiology. Taken together, the patient’s exposure history, normal earlier liver assessments and systematic exclusion of other causes made TCM/NHPs-related DILI progressing to cirrhosis the most coherent explanation.^[7]^

In previous studies, hyperammonaemia was the principal focus of the pathogenesis in AHD; however, increasing evidence indicates that manganese accumulation plays a more crucial role. In patients with cirrhosis and portosystemic shunting, the biliary excretion of manganese is impaired, allowing manganese to bypass hepatic clearance and accumulate systemically. Because the basal ganglia express higher levels of metal transport proteins, such as divalent metal transporter 1, metals including manganese are more likely to accumulate in this region and cause damage.^[1,4,22]^ The hyperintense signal observed on T1-weighted brain MRI further supports this mechanism, as it is regarded as a characteristic change resulting from metal deposition.^[23]^ In our case, marked hypermanganesemia (> 2 × the upper limit of the normal range), together with characteristic T1 MRI findings and compatible neurological manifestations, provided supporting evidence for this mechanism. It is noteworthy that this pattern differs from occupational manganism, which typically occurs in the absence of liver dysfunction.^[1]^

In terms of treatment, it should be emphasized that AHD currently lacks a standardized therapeutic guideline, with management largely focused on the underlying liver disease and providing symptomatic relief. In this case, the patient did not present with any overt primary liver disease; therefore, identifying her history of TCM and NHPs use and advising her to discontinue these agents were particularly important. Currently, there are no established guidelines or consensus on the management of hypermanganesemia. Chelation agents such as sodium dimercaptosulfonate and DMSA were originally developed for heavy-metal intoxication.^[24,25]^ Based on our extensive experience in treating Wilson disease, we prescribed DMSA in this case for manganese chelation given its affinity for divalent metals, established use in heavy‑metal intoxication, and expected efficacy in lowering systemic manganese burden and improving neurological manifestations.^[26,27]^ This case provides a potential therapeutic paradigm for patients with AHD. For the management of extrapyramidal symptoms, this patient was dominated by postural and intention tremor rather than the classic resting tremors of Parkinson disease. Consequently, classic levodopa has not been prioritized. Instead, clonazepam and trihexyphenidyl hydrochloride were applied.^[28,29]^

This case report has several important clinical implications. First, clinicians encountering unexplained extrapyramidal symptoms should not overlook liver screening. Second, clinicians evaluating unexplained liver diseases are strongly advised to routinely enquire about TCM and NHPs use. These products are not always benign and may cause both direct hepatic injury and secondary neurological syndromes. Third, in cirrhotic patients presenting with extrapyramidal symptoms, diagnostic evaluation should not be limited to ceruloplasmin, 24-hour urinary copper excretion, or serum iron metabolism parameters. Whole-blood manganese measurement and careful assessment of pallidal T1 signal on MRI are essential to avoid misdiagnosis. For oncologists, these steps are particularly important because supplement-related liver injury may compromise the hepatic reserve and alter the metabolism, tolerability, and safety of anticancer therapies. Fundamentally, in regions or communities with a long tradition of herbal or alternative medicine use, multidisciplinary management involving neurologists, hepatologists, oncologists, and pharmacologists or toxicologists should be actively involved to ensure early recognition and prevention of such complications.^[1,30,31]^

This study had several limitations. The patient declined liver biopsy, precluding histological confirmation of the liver injury. In addition, precise substance identification and toxicological analysis of all consumed TCM and NHPs could not be completed. Given these limitations, our case relied more on probabilistic reasoning and clinical exclusion rather than definitive evidence. Nevertheless, combined with a long and temporally relevant exposure history, objective evidence of cirrhosis and portosystemic shunting, characteristic MRI findings, and marked hypermanganesemia, this remains a highly instructive and valuable case. This is particularly meaningful in patients with coexisting oncological conditions, where unrecognized TCM/NHPs-related hepatotoxicity may delay treatment, limit therapeutic options, or be misattributed to anticancer agents. For neurologists, this case underscores the importance of considering TCM/NHPs-related hepatotoxicity and manganese toxicity in patients with unexplained tremor, since overlooking these factors may hinder accurate diagnosis and timely management.

## Acknowledgments

We thank the patient and her family for their cooperation and support.

## Author contributions

**Conceptualization:** Chuan Liu, Wang Ni, Gonglu Liu, Yi Dong.

**Data curation:** Chuan Liu, Xiaoyan Li, Wang Ni, Gonglu Liu, Yi Dong.

**Formal analysis:** Chuan Liu, Yi Dong.

**Funding acquisition:** Roumin Wang.

**Investigation:** Chuan Liu, Roumin Wang, Xiaoyan Li, Wang Ni, Gonglu Liu, Yi Dong.

**Methodology:** Chuan Liu, Xiaoyan Li, Wang Ni, Gonglu Liu, Yi Dong.

**Project administration:** Yi Dong.

**Resources:** Chuan Liu, Roumin Wang, Xiaoyan Li, Wang Ni.

**Software:** Chuan Liu, Roumin Wang.

**Supervision:** Yi Dong.

**Validation:** Chuan Liu, Roumin Wang, Wang Ni, Gonglu Liu, Yi Dong.

**Visualization:** Chuan Liu, Roumin Wang, Xiaoyan Li, Wang Ni.

**Writing – original draft:** Chuan Liu.

**Writing – review & editing:** Yi Dong.
